# Editorial: Methods and applications in cardiac electrophysiology–application to inherited arrhythmias

**DOI:** 10.3389/fphys.2024.1388433

**Published:** 2024-03-06

**Authors:** Nathalie Neyroud, Isabelle Baró, Ange Maguy

**Affiliations:** ^1^ Sorbonne Université, INSERM, Research Unit on Cardiovascular and Metabolic Diseases, UMRS-1166, Paris, France; ^2^ Nantes Université, CNRS, INSERM, L’institut du thorax, Nantes, France; ^3^ Department of Physiology, University of Bern, Bern, Switzerland

**Keywords:** inherited arrhythmias, channelopathies, cardiomyopathies, electrophysiology, ECG

Sudden cardiac death and arrhythmias are rare yet significant complications associated with numerous cardiovascular diseases, posing a substantial global health concern, as they contribute to 15%–20% of total deaths (Hayashi et al., 2015). While acute or chronic coronary heart disease are the most frequent primary causes, genetic disorders like inherited channelopathies or non-ischemic cardiomyopathies predominantly precipitate sudden death in young people (Bagnall et al., 2020). Despite the use of antiarrhythmic treatments, which are often non-specific, early resuscitation and defibrillation, remain the key to survival for these patients.

While genetics and genomics have made immense progress in identifying numerous variants in the DNA of patients suffering from arrhythmias, functional characterization of these variants is essential for a better understanding of the pathophysiology of inherited cardiac arrhythmias and their underlying mechanisms (Schwartz et al., 2020).

Cardiac electrophysiology, a discipline at the intersection of cardiology, physiology, and technology, has improved our knowledge and management of arrhythmic disorders. From its beginnings with the invention of the ECG to the contemporary era of sophisticated mapping systems, the field has witnessed exponential growth, revolutionizing our approach to inherited arrhythmias. By mapping the heart’s electrical activity and unraveling aberrant conduction pathways, cardiac electrophysiology aims not only to elucidate the underlying mechanisms of arrhythmias but also to guide therapeutic interventions, such as catheter ablation (Gorenek et al., 2020).

In parallel, the development of cellular electrophysiology techniques has enabled fundamental research to make enormous progress in understanding the mechanisms that trigger arrhythmias. Recent advances in cell-based models, in engineered heart tissues and in animal models of inherited arrhythmias have improved our understanding of pathological remodeling, arrhythmia mechanisms and drug effects and led to major developments in therapies (Odening et al., 2021).

In addition to review the variety and complexity of methodological approaches for the assessment of cardiac electrophysiology to study inherited arrhythmias, this Research Topic aims to enlighten their challenges and limitations. This series of 7 articles highlights some of the latest experimental techniques and methods used to understand the most fundamental questions in cardiac electrophysiology research from molecular to organ function in living organisms and their application to inherited arrhythmias.

The origin of the ventricular tachyarrhythmia’s activation is of major importance for the treatment of patients but also to provide meaningful insights into the exact mechanisms involved. In this Frontiers Research Topic, Zhou et al. used patient electrograms to improve the accuracy of their previously published approach using the smallest angle between two vectors algorithm (SA) to localize the origin of the ventricular tachyarrhythmia. They conclude that the K-nearest neighbors (KNN) algorithm reduces projection errors and therefore improves the localization accuracy of the arrhythmic foci. Their work will help refine existing non-invasive methods and improve our understanding of inherited ventricular arrhythmias.

In the same context, Stoks et al. compare the two most commonly used methods for determining repolarization time on the intracardiac unipolar electrogram with the aim to determine the optimal method in Langendorff-perfused pig hearts, and conclude that the Wyatt method could unify and facilitate repolarization assessment and amplify its role in basic and clinical electrophysiology. Given that many arrhythmia syndromes are caused by local heterogeneities of repolarization, their findings are likely to improve our knowledge of inherited arrhythmias.

Transgenic mice are invaluable models to decipher the mechanisms of inherited arrhythmias. Recent advances in molecular tools such as CRISPR-Cas and prime editing combined with more accessible genetic testing will certainly lead to a growing interest in such approaches for the study of genetic mutations. In this Research Topic, Ferrand et al. provide guidelines to perform a thorough intracardiac electrophysiological study and assess the susceptibility to ventricular arrhythmias in mice, using transgenic models of laminopathy and Brugada syndrome.

At the single-cell level, Ma et al. address a crucial question concerning the exponential increase in the number of genetic variants and the necessity of evaluating their functional significance using high-throughput approaches. Their review provides a concise yet comprehensive history of the patch-clamp technique. In addition, the authors shed light on the future of functional-variant screening by effectively describing the automated patch clamp approach and highlighting the importance of stringent quality controls to avoid misinterpretation.

Human iPSC-CM cell model is now widely used in electrophysiological investigations. It represents a unique patient-specific model but its lack of maturity is associated with small hERG expression. hERG is responsible for the rapid component of the delayed rectifier K^+^ current I_Kr_ and is the off-target of many drugs. In the drug development process, safety tests on hERG are mandatory. Taking advantage of the use of Cs^+^ also carried by hERG, Bloothooft et al. established a simple protocol for high throughput drug tests on significantly larger hERG-related Cs^+^ currents in hiPSC-CMs using automated patch clamp.

The use of hiPSC-CMs will undoubtedly contribute to the development of precision medicine to provide better care for patients suffering from arrhythmogenic cardiomyopathy, an inherited disease linked to mutations in genes encoding desmosomal proteins, for which there is no specific therapy and whose pathophysiology is poorly understood. In this Research Topic, Reisqs et al. provide a thorough review of the main features of this pathology, the signaling pathways involved in disease progression and the studies published on arrhythmogenic cardiomyopathy using hiPSC-CMs.

Finally, Verkerk and Wilders did an *in vitro* and *in silico* study to show the advantage of using the dynamic clamp technique for electrophysiological investigations of hiPSC-CMs carrying mutations from patients suffering from hereditary arrhythmias, involving sodium current or Ca^2+^ homeostasis impairment, or to study resting membrane regulation. This technique allows to inject a current with the characteristics of the inward rectifier potassium current I_K1_, poorly active in this model, in order to record more adult-like polarized action potentials during patch-clamp experiments. They demonstrated that this technique should be widely used on hiPSC-CMs, while awaiting the ultimate fully mature hiPSC-CMs.

In conclusion, the convergence of cutting-edge methodologies in cardiac electrophysiology, encompassing electrocardiology, electrophysiological studies and human cell models, heralds a new era of precision medicine in the management of inherited arrhythmias. Given the complexity of these familial cardiac disorders, the integration of these innovative approaches offers hope, paving the way for personalized diagnostic and therapeutic strategies that promise to revolutionize patient care and improve outcomes in the field of inherited arrhythmias.
